# Patterns of antimicrobial resistance in intensive care unit patients: a study in Vietnam

**DOI:** 10.1186/s12879-017-2529-z

**Published:** 2017-06-15

**Authors:** Giang M. Tran, Thao P. Ho-Le, Duc T. Ha, Chau H. Tran-Nguyen, Tuyet S. M. Nguyen, Thao T. N. Pham, Tuyet A. Nguyen, Dung A. Nguyen, Hoa Q. Hoang, Ngoc V. Tran, Tuan V. Nguyen

**Affiliations:** 1 ICU, Gia Dinh People’s Hospital, 1 No Trang Long Street, Binh Thanh District, Ho Chi Minh City, Vietnam; 2 Centre for Health Technologies, University of Technology Sydney, Sydney, Australia; 3 National Hospital of Can Tho, Can Tho City, Vietnam; 4 University of Medical and Pharmacy, Ho Chi Minh City, Vietnam; 5 Cho Ray Hospital, Ho Chi Minh City, Vietnam; 6 Garvan Institute of Medical Research, Sydney, Australia; 7 School of Public Health and Community Medicine, University of New South Wale, Sydney, Australia

**Keywords:** Antimicrobial resistance, Intensive care unit, *Acinetobacter*, *Klebsiella*, *Pseudomonas aeruginosa*

## Abstract

**Background:**

Antimicrobial resistance has emerged as a major concern in developing countries. The present study sought to define the pattern of antimicrobial resistance in ICU patients with ventilator-associated pneumonia.

**Methods:**

Between November 2014 and September 2015, we enrolled 220 patients (average age ~ 71 yr) who were admitted to ICU in a major tertiary hospital in Ho Chi Minh City, Vietnam. Data concerning demographic characteristics and clinical history were collected from each patient. The Bauer–Kirby disk diffusion method was used to detect the antimicrobial susceptibility.

**Results:**

Antimicrobial resistance was commonly found in ceftriaxone (88%), ceftazidime (80%), ciprofloxacin (77%), cefepime (75%), levofloxacin (72%). Overall, the rate of antimicrobial resistance to any drug was 93% (*n* = 153/164), with the majority (87%) being resistant to at least 2 drugs. The three commonly isolated microorganisms were *Acinetobacter* (*n* = 75), *Klebsiella* (*n* = 39), and *Pseudomonas aeruginosa* (*n* = 29). *Acinetobacter baumannii* were virtually resistant to ceftazidime, ceftriaxone, piperacilin, imipenem, meropenem, ertapenem, ciprofloxacin and levofloxacin. High rates (>70%) of ceftriaxone and ceftazidime-resistant *Klebsiella* were also observed.

**Conclusion:**

These data indicated that critically ill patients on ventilator in Vietnam were at disturbingly high risk of antimicrobial resistance*.* The data also imply that these *Acinetobacter, Klebsiella*, and *Pseudomonas aeruginosa* and multidrug resistance pose serious therapeutic problems in ICU patients. A concerted and systematic effort is required to rapidly identify high risk patients and to reduce the burden of antimicrobial resistance in developing countries.

## Background

Antimicrobial resistance is becoming a global health challenge that threatens to disengage many medical achievements of the past century. Since 1940s when antibiotics were introduced into medicine, the health and well-being of people worldwide has significantly improved. However, after many decades of success with antibiotics, the world is facing a serious threat of bacterial infections and antibiotic resistance which is present in all countries in the world, contributing to the global specter of a “post-antimicrobial era”. Apart from resistance in malaria, other resistance in *Klebsiella pneumoniae, E. coli* is also highly prevalence in developing countries. Extensive drug-resistant tuberculosis (i.e., resistant to at least 4 of the core anti-tuberculosis drugs) has been found in 105 countries [1]. In the US alone, the annual cost associated with antimicrobial resistance was estimated to be $55 billion [2]. In the face of such problems, in September 2016, the United Nations General Assembly convened a high-level conference to solve the problem of antimicrobial resistance [3].

Among healthcare settings of high-risk infection, intensive care unit (ICU) is considered an “epicenter of infections”. Patients in ICU are vulnerable to infections because they are exposed to a variety of invasive procedures, including intubation, mechanical ventilation, and vascular access. Moreover, some drugs (sedatives, muscle relaxants) commonly used for ICU patients also increase the risk for infection. In a multicenter study in Europe, between 20 and 30% of ICU admissions had nosocomial infections [4, 5]. Another prospective study in 1265 ICUs from 75 countries found that hospital-acquired infection was present in ~50% of ICU patients [6]. Taken together, these studies clearly indicate that ICU patients are at high-risk for hospital acquired infections, and ICU is an ideal setting for studying the pattern of antibiotic resistance.

Although the vast majority of antibiotic resistance burden is in developing and poor countries, the prevalence and patterns of resistance have not been well documented for these countries. Vietnam is a developing country with a population of 90 million with a heavy demand for healthcare. As far as we know, there have been no studies on the prevalence of and risk factors for antibiotic resistance among ICU patients in Vietnam. In this study we sought to identify patterns of antibiotic resistance in patients admitted to ICU of a major hospital in Vietnam. The finding from this study can contribute to the development of strategies for a better use of prophylactic and empiric antibiotic therapy in ICU patients in developing countries.

## Methods

### Study design and setting

The Study was conducted at Gia Dinh People’s Hospital (GDPH), Ho Chi Minh City, Vietnam. The Hospital is a major tertiary teaching hospital of Ho Chi Minh City, providing care to residents of South Vietnam. The Hospital has a history of more than 100 years of existence, and currently has 1500 beds, but it is also constantly overloaded with patients. The Hospital has 7 intensive care units (ICU), including internal medicine, surgery, coronary events, cardiology, stroke, and neonatal care. The medical ICU (MICU) has 20 beds, with average admission being 5–7 new patients per day (i.e., about 2500 patients a years). The surgical ICU (SICU) has 65 beds and admits about 19,000 patients a year. The stroke ICU has 6 beds and admits patients from emergency room and stable medical ICU patients. The present study had been conducted in ICUs between November, 2014 and September, 2015. The study procedure and protocol were approved by the ethics committee of the University of Medicine and Pharmacy at Ho Chi Minh City.

We included all ICU patients who gave informed consent to participate in the study. If patient could not give informed consent, we sought substituted consent from the patient’s next of kin. Patients on mechanical ventilation via the tracheostomy or endotracheal tube for more than 48 h at MICU, SICU and stroke ICU of GDPH were enrolled into the study. Inclusion criteria included chest radiograph showing new diffuse parenchymal infiltrates. Patients aged less than 18 years of age or those were pregnant were not included in the study. The following patient were also excluded from the study: pneumonia before mechanical ventilation, acute myocardial infarction during the first 24 h, and uncontrolled ventricular arrhythmias.

### Data and sample collection

Demographic and clinical data of patients met the inclusion criteria were collected by a structured questionnaire. Apart from information concerning the admission to ICU, age, gender, main cause of admission, medical history, vital signs and Glasgow coma score were ascertained. Routine biochemistry tests were performed which included complete blood count (CBC), blood urea nitrogen, serum creatinine, blood glucose, and blood electrolytes. Electrocardiogram, chest X-ray, arterial blood gases and specialized tests were also performed if there was indication. Patients on mechanical ventilation were initial setting tidal volume 10 ml per kg, frequency 14 per minute, inspired oxygen fraction (FIO2) 100%, inspiratory time 1.2 s, assist control mode and closed suctioning. After 48 h of mechanical ventilation, they were re-evaluated for temperature, character of sputum, oxygen consumption and use of antibiotics.

All specimens were collected at the bed-side, and then transferred to the microbiology laboratory immediately for inoculation on proper culture media within 2 h. Sample collection was collected from all patients by bedside bronchoscopy procedure. A 600 mm length and 50 mm view depth fiberscope (PortaView LF-TP; Olympus Optical Co. Ltd., Tokyo, Japan) was attached to a video camera (Olympus, Tokyo, Japan) and focus with white balance was manipulated until optimum view was achieved. The fiberscope was lubricated with sterile xylocaine jelly 2% (AstraZeneca, Sweden). Midazolam and fentanyl and/or xusamethonium were used in sedation. The fiberscope was then introduced until 2 cm above the carina, where the tube was railroaded into the trachea under vision. Between 2 and 5 ml of bronchoalveolar lavage (BAL) fluid was collected. Samples were then transferred to the microbiology department within 30 min after collection for analysis.

At the microbiology laboratory, the BAL samples were submitted for Gram stain and culture. Aspiration of 0.5 ml of BAL was diluted 1: 10 with sterile saline 0.9% (4.5 ml), agitation with Vortex mixer VX 200, 1000 rpm in 30 s and incubated at 35 °C in 30 min. A 10 μl of sample diluent 10^−1^ was inoculated onto 5% blood agar, chocolate agar *haemophilus spp.* (CAHI) and MacConKey Agar media. Plates were incubated at 37 °C in 5% CO2 for 18–24 h. Methods used for confirmation of identification included examination of colonial morphology and haemolytic characteristics on appropriate agar media, Gram stain, rapid tests (catalase, oxidase, coagulase, bile solubility, spot indole, latex agglutination). A sample was classified as positive if there were more than 10^4^ cfu per milliliter of BAL (i.e. ≥ 1 colonies on either medium from the 10^−1^ dilution).

The detection of antimicrobial susceptibility was performed by standard susceptibility test using the Bauer–Kirby disk diffusion method. Antimicrobial disc contents were as follows: amikacin 30 μg, gentamycin 10 μg, ceftriaxone 30 μg, ceftazidime 30 μg, cefepime 30 μg, cefoperazone 75 μg, cefoperazone/sulbactam 75/30 μg, piperacillin/tazobactam 100/10 μg, ertapenem 10 μg, imipenem 10 μg, meropenem 10 μg, ciprofloxacin 5 μg, levofloxacin 5 μg, doxycyllin 30 μg, vancomycin 30 μg and colistin 10 μg. The classification of in-vitro antimicrobial susceptibility test results was based on criteria of the Clinical and Laboratory Standards Institute guidelines (CLSI, 2014). Control strains were *Acinetobacter baumannii* ATCC 19606, *Pseudomonas aeruginosa* ATCC 27853, *Klebsiella pneumoniae* ATCC 700603, *Escherichia coli* ATCC 25922, *Staphylococcus aureus* ATCC 25923 (Liofilchem, Italy).

### Data analysis

All patients’ data were electronically stored in a relational database system specifically developed for the study. The data were then exported into a spreadsheet for statistical analyses. The prevalence of antimicrobial resistance was estimated as the proportion of positive results over the entire study sample. Multi-drug resistance (MDR) was defined as resistant to at least two antimicrobial agents. We used a network analysis method to visualize the co-occurrence of resistance to drugs. In this method, the line linking between two drugs represents the number of patients who were resistant to both drugs, and the size of each node represents the number of patients who were on a drug. Each line represents at least 5 patients, and the thicker the line the more number of patients. The graph was constructed for each major organism**.** All analyses were conducted using the R statistical software version 3.13 (R Foundation for Statistical Computing, Vienna, Austria) on the Window platform. The network graph was constructed using the “igraph” package.

## Results

The study enrolled 220 patients; among whom, 50% were females. The average age of patients was 71 years (±16.7 SD). A dominant majority (79%) patients were from medical ICU, and the remaining 20% were from surgical ICU (Table 1). In terms of primary diagnosis, approximately 60% of patients had respiratory failure, followed by hypertension (37%), diabetes (26%), chronic kidney disease (16%), heart failure (16%), sepsis shock (11%). The average APACHE II score was 22 (IQR: 18–28). The median number of days mechanical ventilation before enrollment of study and length of stay ICU were 9 (range: 2–83) and 16 (range: 3–135), respectively.

**Table 1:** Clinical characteristics of 220 patients admitted to ICU

Characteristics	Statistics
Age (yrs; mean [SD])	70.7 (16.7)
N (%)	
Gender (men)	109 (49.5)
Admission category	
Medical ICU	174 (79.1)
Surgical ICU	43 (19.5)
Stroke Unit	3 (1.4)
Primary diagnosis on admission	
Respiratory failure	127 (57.7)
Hypertension	80 (36.5)
Diabetes mellitus	57 (25.9)
Chronic kidney disease	36 (16.4)
Cerebral vascular disease	35 (16.0)
Heart failure	34 (15.5)
Sepsis shock	25 (11.4)
Prior medical history	
Hypertension	113 (62.4)
Diabetes mellitus	70 (38.6)
Chronic kidney disease	24 (13.2)
COPD	24 (13.2)
Others^a^	79 (36.0)
Median (IQR)	
No. of days on mechanical ventilation (before enrollment)	9 (6–11.5)
APACHE II score	22 (18–28)
Length of stay ICU (d)	16 (11–25)

^*^COPD: chronic obstructive pulmonary disease

^a^: *n* (%): cancer: 20 [11]; brain hemorrhage: 19 [10]; tuberculosis: 14 [7]; heart failure: 9 [5]; acute myocardial infarction: 7 [4]; ischemic stroke: 4 [2]; gastrointestinal bleeding, urinary tract infection, prostate enlarge: 2 [1]; others: 84 (46)

At the time of admission to ICU, about 50% and 45% patients were being treated with imipenem and levofloxacin, respectively; followed by 30% with cephalosporin 3th generation, ciprofloxacin (27%), piperacillin – tazobactam (23%), meropenem (22%). Other antibiotic such as aminoglycosides, cefoperazone – sulbactam, colistin and vancomycin were also commonly used (Table 2).

**Table 2:** Pattern of antibiotic use at ICU admission

Antibiotics	*N* (%)
Imipenem	103 (50.0)
Levofloxacin	92 (44.6)
Cephalosporin 3th generation	62 (30.1)
Ciprofloxacin	55 (27.0)
Piperacillin – tazobactam	47 (22.8)
Meropenem	46 (22.3)
Aminoglycosides	35 (17.0)
Vancomycin	29 (14.1)
Cefoperazone - sulbactam	26 (12.6)
Metronidazol	18 (8.7)
Colistin	12 (5.8)
Others	38 (18.4)

At the individual patient level, the prevalence of antimicrobial resistance was 93% (*n* = 153/164), with the majority (87%) being resistant to at least 2 drugs. Drug resistance was commonly found in ceftriaxone (88%), ceftazidime (80%), ciprofloxacin (77%), cefepime (75%), levofloxacin (72%). Resistance to colistin was found in ~2% (*n* = 2) patients. Approximately 85% (*n* = 139) were found to be multiple-drug resistance (MDR).

Among patients with ventilator–associated pneumonia, *Acinetobacter* emerged as the major causative agent that accounted for 42% (*n* = 75) of 177 microorganisms isolated from patients (Table 3). The next major causative organism was *Klebsiella* (22%; *n* = 39), followed by *pseudomonas aeruginosa* (16%; *n* = 29). There were 13 patients with multiple infections, and the majority of coinfection was found in *Acinetobacter spp.* and *Pseudomonas aruginosa* (4.5%; *n* = 8).

**Table 3:** Distribution of microorganisms isolated from patients admitted to ICU (*N* = 177)

Organisms	*N* (%)
*Acinetobacter baumannii*	55 (31)
*Acinetobacter spp*	20 (11.3)
*Klebsiella pneumoniae*	38 (21.5)
*Klebsiella spp*	1 (0.56)
*Pseudomonas aeruginosa*	29 (16.3)
*Escherichia coli*	9 (5.0)
*Staphylococcus aureus*	9 (5.0)
*Burkholderia cepacia*	8 (4.5)
*Enterobacter cloace*	2 (1.1)
*Enterobacter spp*	1 (0.56)
*Stenotrophomonas maltophilia*	2 (1.1)
*Haemophilus influenzae*	1 (0.56)
*Serratia marcescens*	1 (0.56)
*Chryseobacterium indologenes*	1 (0.56)

Analysis by major organism and drug (Table 4) revealed a high rate of antimicrobial resistance among the three major pathogens (*Acinetobacter*, *Klebsiella*, and *Pseudomonas*). For example, the rate of *Acinetobacter* resistance to ceftazidime, ceftriaxone, cefepime, piperacilin, imipenem, meropenem, ertapenem, ciprofloxacin, and levofloxacin was greater than 90%. High rate of resistance to *Klebsiella* was also noted for ceftriaxone (83%), ceftazidime (76%), and cefoperazone (73%). Among the 29 isolates of *Pseudomonas*, drug resistance was found in meropenem (86%), imipenem (79%), gentamycin (80%), ciprofloxacin (80%), and levofloxacin (76%). Even colistin was found to be resistant to *Acinetobacter* (1.5%) and *Pseudomonas* (3.4%).

**Table 4:** Antibiotic resistance of Gram-negative microorganism isolated from ICU patients

Antibiotic drug	*Acinetobacter* *n* = 75	*Klebsiella* *n* = 39	*Pseudomonas* *n* = 29
Cefoperazone	Not done	73.3	Not done
Ceftazidime	93.2	76.3	72.4
Ceftriaxone	95.2	82.7	100
Cefoperazone-sulbactam	4.3	21.0	60.0
Cefepime	93.6	65.7	61.9
Piperacilin - Tazobactam	95.0	64.1	32.1
Imipenem	93.2	25.6	79.3
Meropenem	90.5	20.0	86.2
Ertapenem	100.0	46.4	Not done
Amikacin	77.8	5.1	65.5
Gentamycin	84.1	27.8	80.0
Ciprofloxacin	95.2	52.6	80.0
Levofloxacin	91.5	56.7	75.8
Colistin	1.5	Not done	3.4

Numbers shown are percent of organism-specific total

**Fig. 1:**
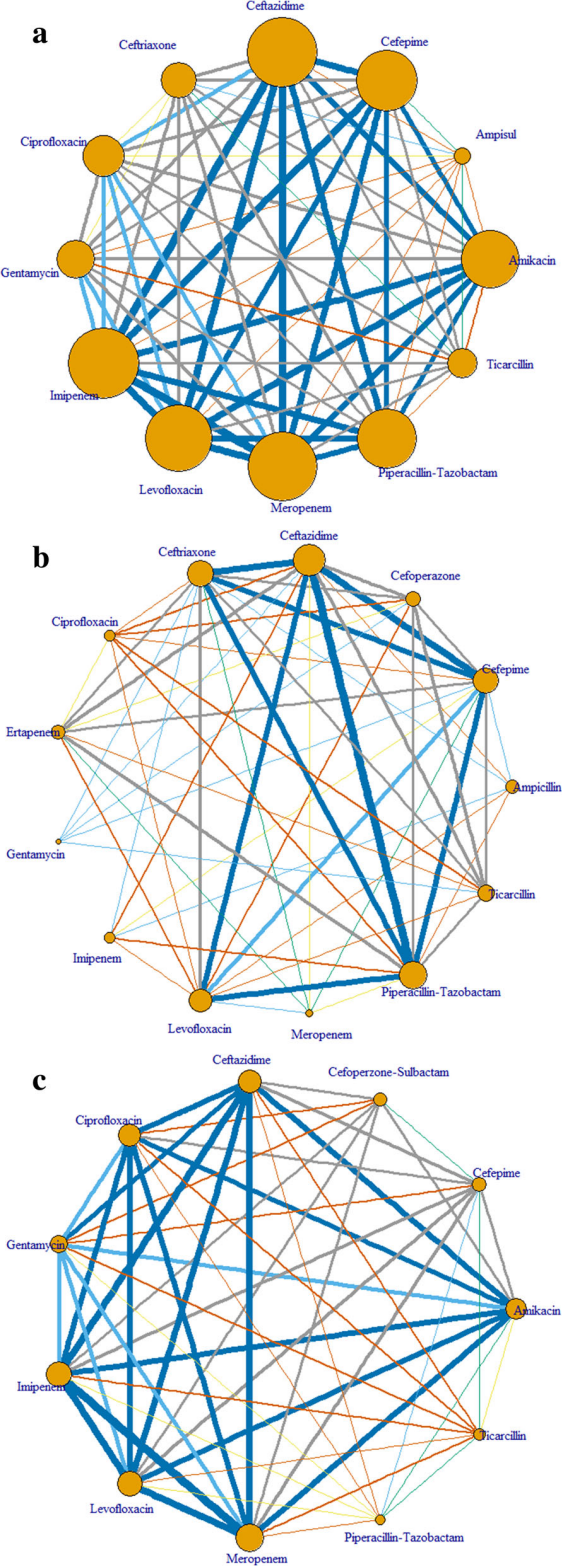
Network graph of multidrug resistance in *Acinetobacter* (panel **a**), *Klebsiella* (panel **b**) and *Pseudomonas* (panel **c**). The size of each node proportionally represents the number of patients on a drug. The line linking between any two antibiotics represents at least 5 patients. Thus, each line also represents the magnitude of multidrug resistance

The patterns of antimicrobial resistance in *Acinetobacter*, *Klebsiella,* and *Pseudomonas* formed a network of multiple drugs as depicted in Fig. 1. In this figure, the degree of thickness of the connecting lines represents the strength of association between any two drugs. For instance, *Acinetobacter* was resistant to ceftazidime, ceftriaxone, cefepime, ertapenem, and ciprofloxacin, and these resistant pathways formed a complex network. Similar complex networks of drug resistance were also observed for *Klebsiella* and *Pseudomonas*.

During the follow-up period, 149 patients had died (incidence rate 68%). The risk of death was associated with advancing age (*P* = 0.01) and shorter length of ICU stay (*P* = 0.01). On average, the deceased group had a greater APACHE II score than survivors (23.9 vs 22.1), but the difference was not statistically significant (*P* = 0.08). The risk of mortality among patients with drug resistance was 67% (103/153) compared with the risk among patients without drug resistance (45%; 5/11); however, the difference did not reach a statistical significance (*P* = 0.19, Fisher’s exact test). Approximately 91% of those who died in the drug resistance group were MDR. The risk of mortality among patients infected with *Acinetebacter*, *P. aeruginosa,* and *Klebsiella* spp. was 67%, 60%, and 55%, respectively, but there was no statistically significant difference between the three organisms.

## Discussion

Drug resistance, including antimicrobial resistance, is a global issue, because it affects people all over the world, particularly people in developing countries. Based on a sample of ICU patients in a major hospital in Vietnam, we have shown that approximately 95% of ventilator-associated pneumonia patients were resistant to antibiotic drugs. More disturbingly, ~85% (*n* = 139) were multiple-drug resistant. These results deserve some further elaboration.

In this study, we found that the distribution of pathogens was different from previous studies in developing and developed countries. In most Asian countries, common pathogens isolated from ICU patients were *P. aeruginosa, Klebsiella* spp., *E. coli, Enterococcus*, and *Staphylococcus aureus* [7]. Moreover, the Canadian National Intensive Care Unit study found that *P. aeruginosa, Staphylococcus aureus, Haemophilus influenzae, Enterococcus spp.*, *Staphylococcus pneumoniae*, and *K. pneumoniae* are the most common isolates [8]. However, in our study, the most common pathogen was *Acinetobacter,* followed by *Klebsiella* and *P. aeruginosa.* Both *Acinetobacter* and *P. aeruginosa* demonstrated multidrug resistance to several antibiotics.

A striking finding from this study was the degree of drug resistance among key pathogens. We found a very high rate of resistance (>70%) among *Acinetobacter* isolates to most antibiotics, except cefoperazone-sulbactam (4.3%) and colistin (1.5%). Among *Klebsiella* isolates, low resistance was found for amikacin (5.1%), but high for cefoperazone, ceftazidime, ceftriaxone, cefepime, and levofloxacin. Resistance to meropenem, imipenem, gentamycin, ciprofloxacin and levofloxacin among *P. aeruginosa* was consistently over 80%. This is probably due to the extensive use of third generation of cephalosporins and quinolone antibiotics in ICU patients.

Previous studies found that the opportunistic pathogens *Acinetobacter spp.,* mainly *A. baumannii,* are commonly found in ICUs. Our study also found that *Acinetobacter* was the most common pathogen in ICU patients, and this finding has significant clinical implications. This *Acinetobacter spp.* has shown a remarkable resistance to many antibiotic classes. *Acinetobacter* infection is also associated with a high risk of mortality, as there are limited treatment options for this infection. In our study, apart from colistin, *Acinetobacter* was resistant to virtually all antibiotics, including imipenem (93%), meropenem (90%), ertapenem (100%), ceftriaxone (95%), ceftazidime (93%), cefepime (94%), piperacillin (95%), and ciprofloxacin (95%). Therefore, *Acinetobacter* is increasingly recognized as an important cause of hospital-acquired infection [9, 10], and our finding confirms the significance of this species as a leading cause of MDR infection in critically ill patients with ventilator-associated pneumonia.

*Klebsiella* is considered an opportunistic pathogen, and *Klebsiella* infection is commonly found in hospitalized patients with conditions such as diabetes and chronic pulmonary obstruction [11]. In human, *K*. *pneumoniae,* the most important species of the genus, was identified as a pulmonary pathogen more than 100 years ago. While *K*. *pneumoniae* has been declining in the United States [12], it is still common in China [13], particularly in ventilator-associated pneumonia patients [14]. In this study, we found that the pathogen accounted for ~22% of all infected patients, and the pathogen is also common in patients with diabetes and pulmonary disorders. In Southeast Asia, among *K*. *pneumoniae* isolates, the resistance rate to ciprofloxacin was 62% in Philippines, 29% in Thailand, and 22% in Singapore [15]. In our study, the rate of resistance to ciprofloxacin was 53%. It is not clear why there exists geographic differences in the rates of resistance between countries, but the interaction between environmental reservoir and host variables may be a contributory factor.

In this study *P. aeruginosa* was the third most common pathogen in ventilator-associated pneumonia. Previous studies have also observed that *P. aeruginosa* is a prominent cause of pneumonia in hospital setting in Southeast Asia. In previous studies, ciprofloxacin resistance rates among *Pseudomonas spp.* were over 10% in Malaysia and Singapore [15], which was much lower than our study’s rate (80%). Although the infection may be due to reservoirs within the hospital or ICU environment, selective antimicrobial pressure and compromise of the respiratory tract can also be a risk factor.

Our findings have important clinical implications in the treatment and management of ICU patients, particularly those with ventilator-associated pneumonia. First, clinicians should realize that there is a high possibility that ventilator-associated pneumonia patients are infected with the three common pathogens, and that multiple drug resistance is a reality. Second, the high rate of multidrug resistance observed in this study is a serious concern in the management of ICU patients. It calls for a more systematic approach to reduce antibiotic resistance rates, and minimizing the use of broad-spectrum antibiotics. Third, in the presence of multidrug resistance, the development of rapid diagnostic test for prompt targeted therapy is an important priority. There is also a need for implementing a drug monitoring system that optimizes drug administration and enable a more personalized approach to treatment.

The present study’s findings nevertheless should be interpreted within context of strengths and weaknesses. The study was conducted in a well characterized cohort of patients, who had been thoroughly followed. We have been able to analyze a whole spectrum of pathogens and antibiotic treatments, which allow a relatively comprehensive documentation of antimicrobial resistance in ICU patients. However, we did not have the capacity to conduct a 16S–rRNA gene sequence analysis to identify bacterial species, and this is a significant weakness of the study. Moreover, we did not undertake a phenotypic test that would provide an insight into the mechanism of drug resistance. The study was based on a tertiary hospital and the data were limited to ICU patients; thus the findings may not represent the community acquired infection in Vietnam. The sample size for the study was modest, and did not have adequate power to detect smaller effect sizes or rare incidence. We did not ascertain the specific causes of infection and comorbidities. We also did not ascertain all aspects of care that may have resulted in the prescription of inappropriate antibiotics in our patients.

## Conclusion

In this first hospital based study in Vietnam, we observed that ICU-acquired *Acinetobacter, Klebsiella* and *P. aeruginosa* predominate ventilator-associated pneumonia patients. More disturbingly, ~85% were multiple-drug resistant. The finding here reinforces the view that multidrug resistance is a global public health issue, and emphasizes the need to study combined therapies and rational treatment strategies.

## Abbreviations

APACHE II: Acute Physiology and Chronic Health evaluation II
ATCC: American Type Culture Collection
cfu: colony forming unit
GDPH: Gia Dinh People’s Hospital
ICU: Intensive Care Unit
IQR: Interquartile Range
